# Clinical, laboratory and radiological assessment of skeletal maturation in children and adolescents with obesity

**DOI:** 10.1186/s43054-020-00024-0

**Published:** 2020-05-04

**Authors:** Rania S. M. Ibrahim, Christine William Shaker, Marwa Farouk Mira, Marwa Ahmed Sedky, Ghada Mohammad Anwar

**Affiliations:** 1 Radiology Department, Faculty of Medicine, Cairo University, Kasr Al-aini Street, Al-manial District, Cairo, 12652 Egypt; 2 Cairo, Egypt; 3 Diabetes Endocrine and Metabolism Pediatric Unit, Pediatric Department, Faculty of Medicine, Cairo University, Kasr Al-aini Street, Al-manial District, Cairo, Egypt

**Keywords:** Skeletal maturation, Obesity, Bone age, Children, Adolescents, Puberty

## Abstract

**Background:**

Childhood obesity is related to multiple serious health problems and an enhanced risk of premature onset of diseases. The relation of skeletal maturation to obesity is undetermined. The study aims to evaluate skeletal maturation in children and adolescents with obesity, to correlate it with their anthropometric data, body fat content, BMI, fat mass and pubertal stage.

**Results:**

Our study shows that obese children and adolescents have accelerated skeletal maturation compared to control normal-weight healthy subjects (mean skeletal age difference of 0.123 ± 0.67 years versus − 0.175 ± 0.32 years). Also, there were significant positive correlations between bone age and BMI (*r* = 0.435, *P* value 0.00).

**Conclusion:**

The mean skeletal age difference was more in the obese group as compared to the control group and suggesting accelerated skeletal development in the obese group. It is important to assess skeletal maturity in growing patients to determine the best timing for orthopedic and orthodontic treatment around the growth spurt.

## Background

Recently, the overweight and obesity in children’s prevalence are steadily increasing worldwide. The World Health Organization has outlined obesity as “a world epidemic disease” [1]*.* Childhood obesity is related to multiple serious health problems and an enhanced risk of premature onset of diseases, including diabetes and heart disease [2]*.* Skeletal maturity is used to measure the development depending on bone size, shape, and degree of its mineralization to determine its closeness to full maturation [3]. In general, females have advanced bone age compared to boys. The distinction is present at birth and persists along the growth period; however, it is more noticeable after the beginning of puberty [4].

The hand and wrist radiographs are appropriate for assessment of the bone age because it is simple, and it possesses several bones. Therefore, the most ordinarily utilized in clinical practice are the atlas-based technique of Greulich and Pyle. The radiographs are performed in chronologic steps up to 19 years in boys and 18 years in girls. The simplicity and speed with which a skeletal age can be assigned have created this atlas the foremost ordinarily used customary used of reference for skeletal maturation worldwide [3]. Bone age (BA) is compared with the chronological age (CA). A distinction between these two values indicates abnormalities in skeletal development. It is usually utilized in the diagnosis and management of endocrine disorders, and it may serve as a sign of the therapeutic result of treatment [4].

Previous studies showed contradictory results concerning the relation of skeletal maturation to obesity [5]. The sole body measurement that directly calculates the relative composition of the body and presents a measure of fitness level, regardless of the height or weight, is body fat percentage (BF%). Wide application of body mass index (BMI) affords a measure that permits a comparison with the overweight subjects of various heights and weights [6]. The level of body fat can affect the neuroendocrine events that are responsible for the onset of premature puberty, despite low levels of the growth hormone. More specific, leptin is the key hormone concerned in this method [7]. Knowledge of skeletal development is important as the patient’s response to the treatment will be more effective if skeletal development has not yet reached its end. Moreover, dentofacial and orthopedic treatment performed after the growth phase is not effective in improving skeletal discrepancies. So it is important to assess skeletal maturity in growing patients to determine the best timing for orthopedic and orthodontic treatment around the growth spurt [8].

## Methods

This is a case-control study conducted at Diabetes Endocrine and Metabolism Pediatric Unit (DEMPU) in collaboration with Pediatric Radiology Department Unit; University Children Hospital. A total of 30 obese children and adolescents compared with similar number of age and sex-matched healthy subjects, who enrolled as controls. This study has been carried out between August 2017 and August 2018. All patients’ parents were counselled and signed a consent form. Inclusion criteria are as follows: children and adolescents between 8 and 15 years, both sexes, BMI levels above the 95th percentile using Egyptian growth curves [9]. and exogenous obesity. Exclusion criteria are as follows: patients with endocrinal disorders, patients with syndromic obesity, patients on drugs causing overweight or skeletal advancement (e.g., steroid, androgens), and subjects with any co-morbid chronic diseases.

All enrolled subjects were subjected to detailed medical history with special emphasis on the demographic data; gender and chronological age, family history of obesity, chronic disease or endocrinal disease, obesity onset, nutritional history, lifestyle, complications: shortness of birth, orthopnea, social isolation, low self-esteem and snoring, and drugs especially steroids and androgens.

Full clinical examination with special emphasis on the anthropometric measurements (weight, height, BMI were obtained and plotted against appropriate Egyptian growth charts). Weight was measured with subjects in minimal light clothing, using a standard balance sensitive to 0.1 kg. Height was determined to the nearest 1 mm using a Harpenden stadiometer. Body mass index was calculated by dividing weight to the square of height (kg/m2). Tanner staging for pubertal assessment was done [10].

Measurements of total body fat contents (kg), total body fat %, lean body mass (kg), lean body mass % using Tanita apparatus for bioelectrical impedance analysis.

Laboratory investigations: Recent lipid profiles (serum cholesterol, HDL, LDL and triglycerides (TG)) were obtained from the files of the patients. The hormonal profile and HbA1c were revised to exclude any endocrinal cause.

**Fig. 1:**
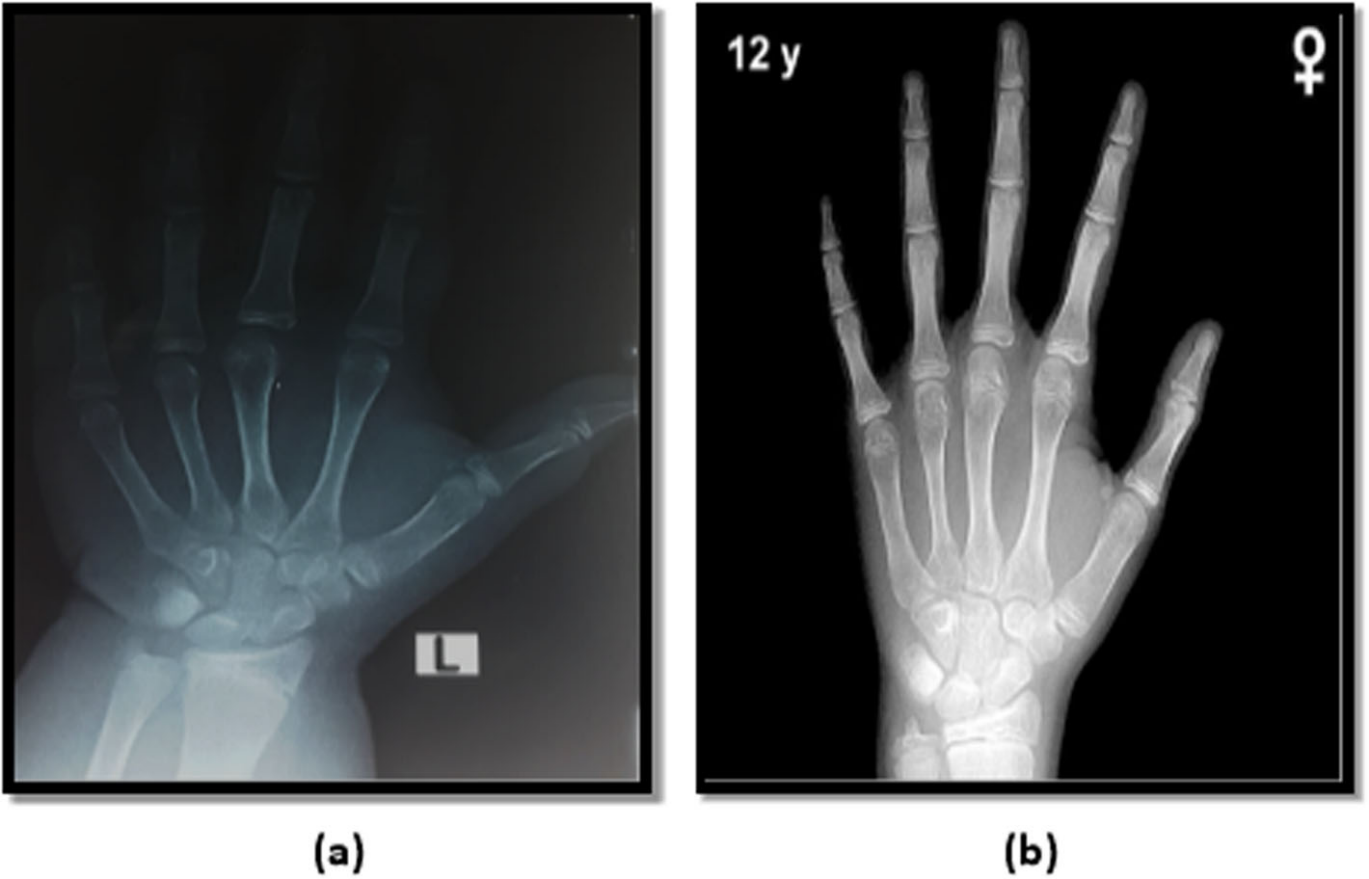
**a** Radiograph of the left hand obtained in an 11.4-year-old Egyptian girl. **b** Greulich and Pyle method atlas image showing advanced bone age (12 years old)

**Fig. 2:**
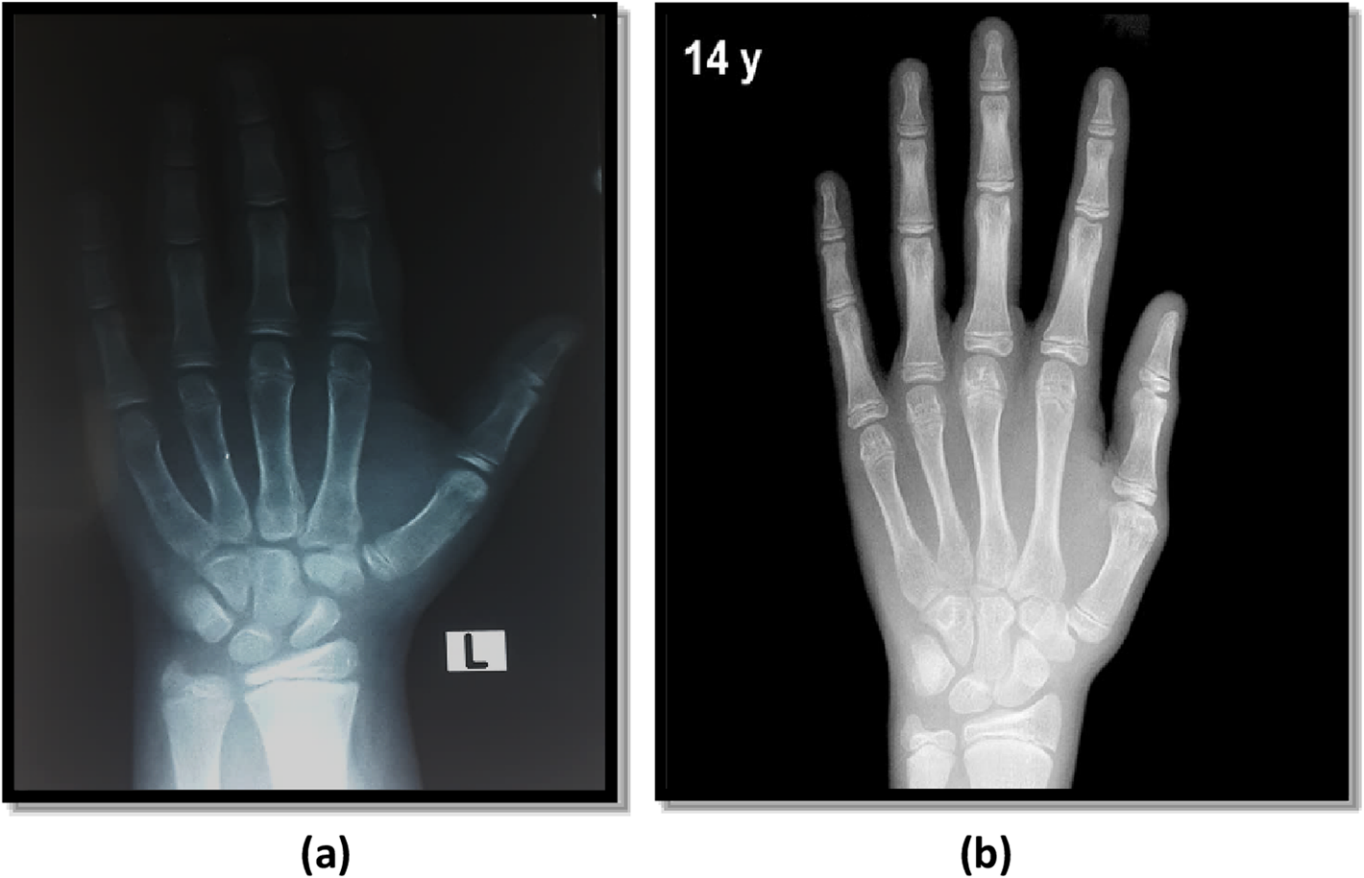
**a** Radiograph of the left hand obtained in a 12.3-year-old Egyptian boy. **b** Greulich and Pyle method atlas image showing advanced bone age (14 years old)

**Fig. 3:**
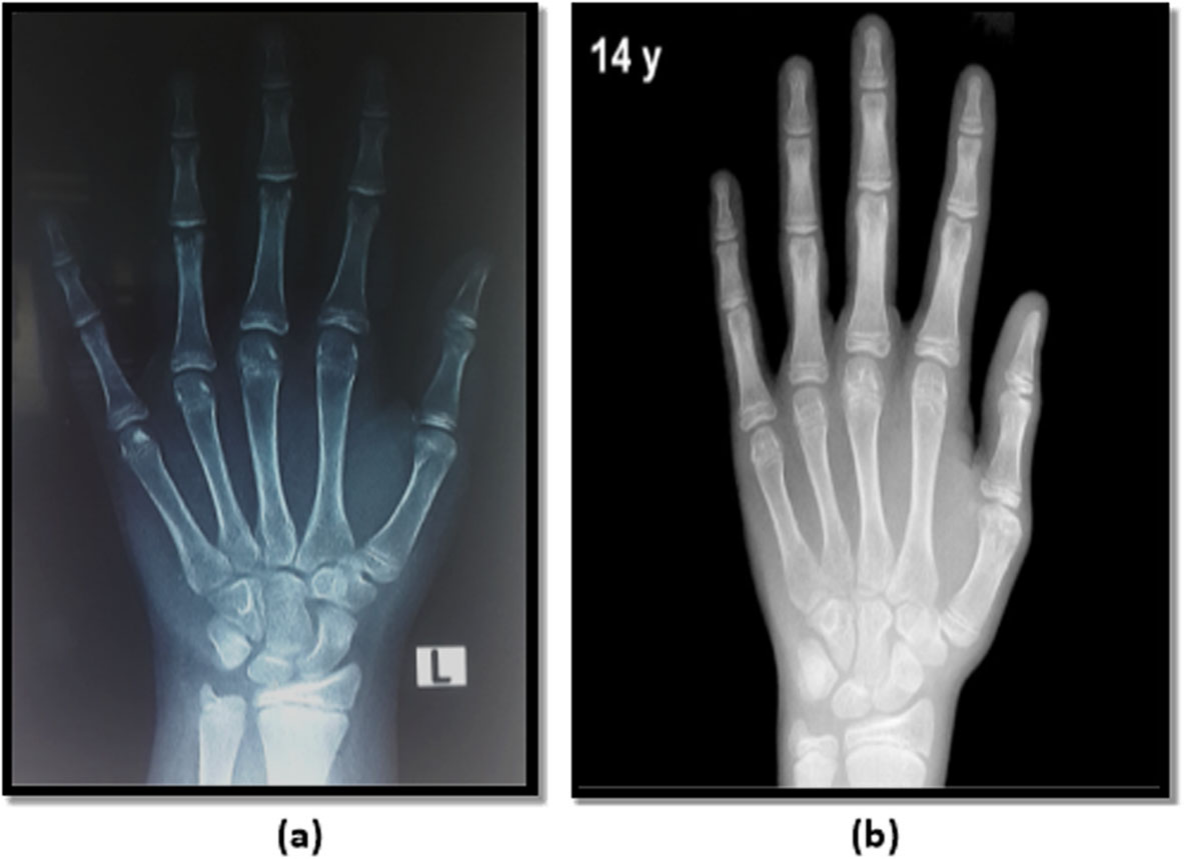
**a** Radiograph of the left hand obtained in a 12.3-year-old Egyptian boy. **b** Greulich and Pyle method atlas image showing advanced bone age (13 years old)

The left hand-wrist radiographs were done for assessing skeletal age using Greulich and Pyle method for skeletal age determination [3]. The radiograph to be assessed is compared with the series of standard plates, and the age given to the standard plate that fits most closely is assigned as the skeletal age of the child. Examples to bone age readings are demonstrated in Figs. 1, 2, and 3.

### Statistical analysis

The data were analysed using the S-plus Statistics Software (SPSS version 21). Descriptive statistical calculations (mean, standard deviation, median) were done to quantitative values, e.g., age, anthropometric measurements, age of diagnosis, and laboratory results. Statistical analysis was performed by comparing each independent variable between case and control group using the independent *t* test, as applicable for quantitative variables and Pearson’s chi-square test for qualitative variables. Two-tailed *P* values of less than 0.05 were considered to be significant. The Pearson correlation coefficient (*r*) was used to express the relationship between quantitative variables in the case and control group. Mann-Whitney test was used to express the relation between laboratory results between case and control. Multivariable regression analysis was used to examine the relation between skeletal maturation; bone age and other variables, as adjusted for the possible confounding effect of variables expected to influence the outcome of interest.

## Results

The study population included 30 obese children and adolescents, 14 males (46.7%) and 16 females (53.3%) with male to female ratio (1: 1.1). Patients were compared with 30 age and sex-matched healthy subjects enrolled as controls. The control group consisted of 15 (50%) males and 15 (50%) females (ratio: 1:1). The mean age of the obese group was 10.07 ± 1.5 years while that of controls was 9.8 ± 1.5 years.

In this study, we found no significant difference in the Tanner stage in females; this may be due to a small sample size; however, in males, there is a significant difference between obese and control groups (Table 1).

**Table 1:** Comparison of Tanner stages in males and females between obese and control

Tanner stage	Male		Tanner stage	Female	
	Obese	Control		Obese	Control
1	7 (50%)	15 (100%)	1	5(31.3%)	7(46.7%)
2	2 (14.3%)	0	2	6(37.5%)	5(33.3%)
3	4 (28.6%)	0	3	2(12.5%)	2(13.3%)
4	0	0	4	2(12.5%)	1(6.7%)
5	1 (7.1%)	0	5	1(6.3%)	0
*P* value	**0.02**		*P value*	**0.786**	

The current study showed that the mean of the percentage of total body fat content in the obese group was 42.83 ± 7 %, accounting for 27.42 ± 12.97 kg of body weight while meaning of lean body mass % was 67.7 ± 9 %, and that of lean body mass was 42.6 ± 11.87 kg (Table 2).

**Table 2:** Body fat versus lean body mass in obese group

Total body fat content %	
Mean ± SD	42.83 ± 7
Range	33–63.6
Total body fat content (kg)	
Mean ± SD	27.42 ± 12.97
Range	15.3–76.3
Lean body mass %	
Mean ± SD	67.7 ± 9
Range	28.7–74.5
Lean body mass	
Mean ± SD	42.6 ± 11.87
Range	28.7–75

The mean cholesterol blood level in the obese group was 170.47 ± 49.22, HDL level was 39.02 ± 8.69, LDL level was 107.03 ± 47.26 while triglycerides level was 111.77 ± 45.15.

The mean skeletal age difference was more in the obese group (0.123) as compared to the control group (− 0.175), and it was statistically significant (*P* = 0.032) (Table 3).

**Table 3:** Comparison of skeletal age difference between obese and control

	Obese	Control	*P* value
Total body fat content %			
Mean ± SD	42.83 ± 7	22.34 ± 3.877	**0.000**
Range	33–63.6	12–30	
Total body fat content (kg)			
Mean ± SD	27.42 ± 12.97	7.76 ± 2.54	**0.000**
Range	15.3–76.3	2.9–13.2	
Lean body mass %			
Mean ± SD	67.7 ± 9	83.25 ± 2.92	**0.000**
Range	28.7–74.5	77.5–88.4	
Lean body mass			
Mean ± SD	42.6 ± 11.87	35.2 ± 11.69	**0.000**
Range	28.7–75	18.9–40.4	

There was a non-significant skeletal age difference between males and females (*P* value 0.138). Lipid profiles in obese and control groups were presented in Table 4.

**Table 4:** Lipid profile in obese versus control group

Variable	Obese group	Control	*P* value
Cholesterol			
Mean ± SD	170.47 ± 49.22	157.97 ± 16.85	**0.554**
Range	98–367	130–195	
HDL			
Mean ± SD	39.02 ± 8.69	49.17 ± 10.68	**0.000**
Range	23–54	33–67	
LDL			
Mean ± SD	107.03 ± 47.26	88 ± 17.7	**0.062**
Range	11–269	42–130	
Triglyceride			
Mean ± SD	111.77 ± 45.15	103.13 ± 15.11	**0.549**
Range	32–223	78–131	

**Fig. 4:**
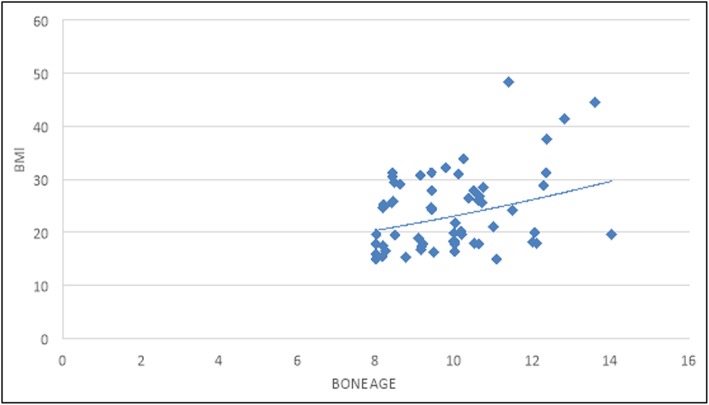
Correlation between bone age and BMI

Our results showed significant positive correlation between bone age and BMI (*r* = 0.435, *P* value = 0.00) (Fig. 4).

**Fig. 5:**
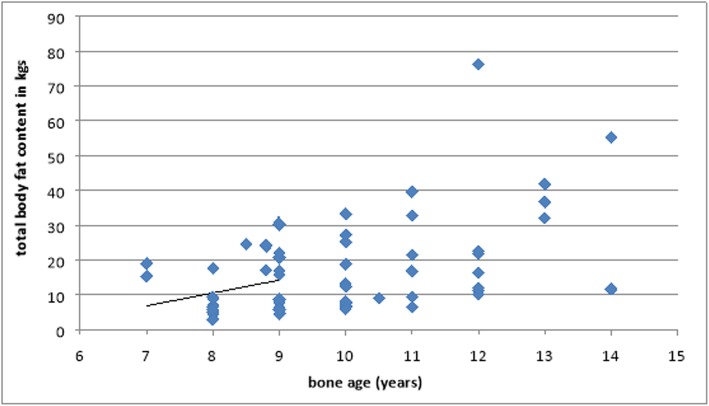
Correlation between bone age and body fat content

**Fig. 6:**
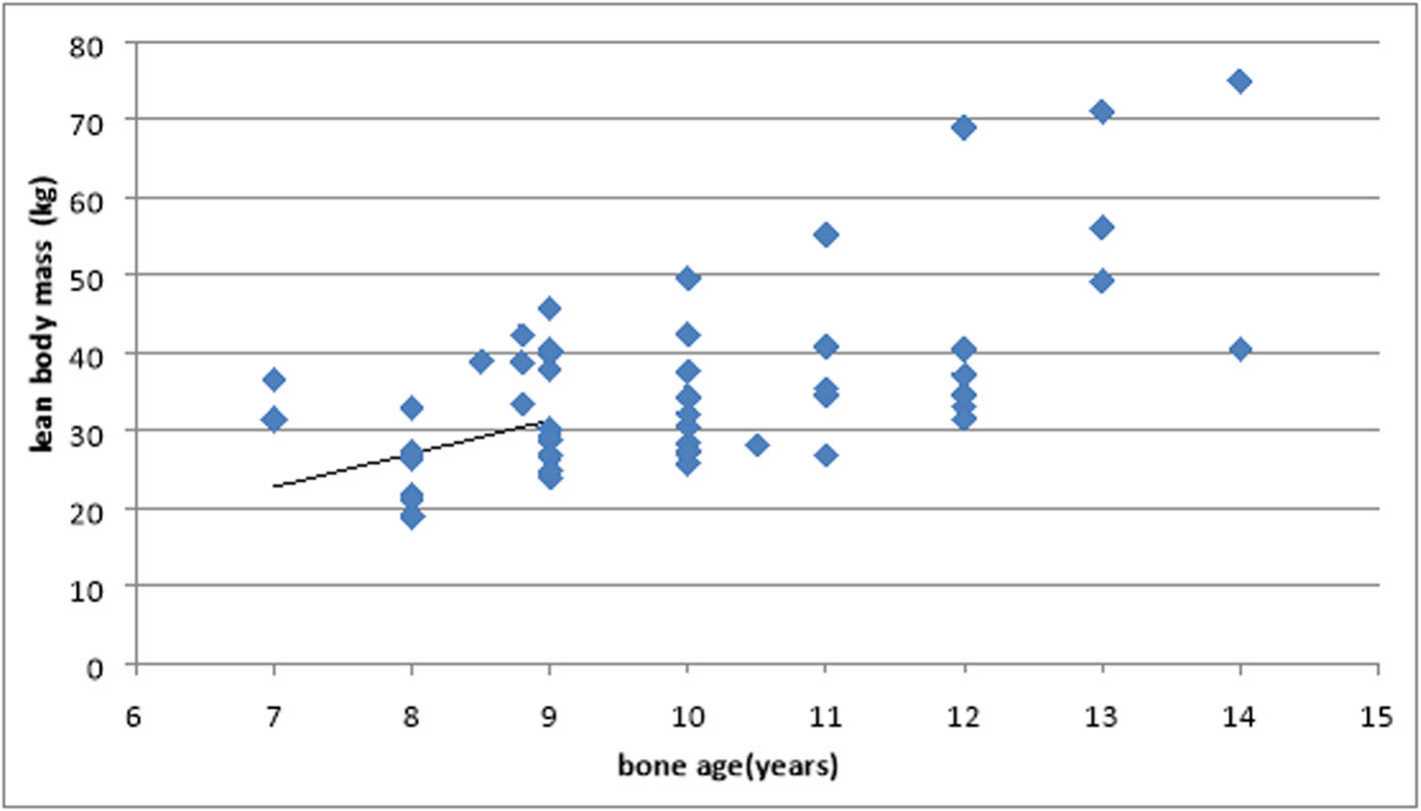
Correlation between bone age and body mass in kilograms

There is significant correlation between bone age and total body fat content (*r* = 0.463, *P* value 0.00), significant positive correlation between bone age and lean body mass (*r* = 0.622, *P* value 0.00), and significant negative correlation between bone age and percentage of lean body mass (*r* = − 0.313, *p* 0.015) (Figs. 5 and 6).

There is no significant correlation between bone age and lipid profile of the patients.

There is a significant positive correlation between bone age and pubertal development stages (*r* = 0.676, *P* value 0.00). There is significant correlation between BMI and pubertal stage (*r* = 0.466, *P* value < 0.05).

Regression analysis was run to select the minimum combination of variables that maximally influence skeletal maturation assessed by bone age. Bone age was used as the dependent variable; the confounders included were as follows: BMI, puberty, lean body mass, total body fat content, and lipid profile. It was revealed that there was one significant predictor for bone age and directly related to it, which is the lean body mass (with OR = 0.129; 95% CI 0.049–0.210, *P* = 0.002), whereas the other confounders showed non-significant relation to the outcome.

## Discussion

Relation of skeletal maturation to obesity was a point of interest to researchers; however, previous studies showed contradictory results about this relation [11].

This study was done to evaluate skeletal maturation and age in obese children and adolescents and correlate it with their chronological age, anthropometric data, and body fat content.

Our study shows that obese children and adolescents have accelerated skeletal maturation compared to control normal-weight healthy subjects (mean skeletal age difference of 0.123 ± 0.67 years versus − 0.175 ± 0.32 years). Also, bone age and BMI showed a significant correlation (*r* = 0.435, *P* value = 0.00).

This can be explained by leptin; made by the adipose tissue once, in high quantity, it will stimulate skeletal growth through the activation of assorted mediators, such as insulin-like growth factor 1 and sex hormones. So an obese subject has a high sensitivity to leptin at a peripheral level, leading to increased differentiation and proliferation of chondrocytes and resulting in precocious skeletal maturation [12].

A study done by Russell et al. 2001 investigated the connection between skeletal maturation and adiposity in American and Caucasian children. Hand-wrist radiographs were accustomed to assess skeletal maturity and age, whereas BMI standard deviation score (SDS) was the measure of adiposity. The findings were prompted that there was a significant correlation between skeletal age and BMI as American children were considerably heavier than Caucasians (BMI SDS 2.7 ± 3.4 vs 1.7 ± 2.4, *P* < 0.05). Both BA–CA (0.75 ± 1.46 vs 0.28 ± 1.38, *P* < 0.05), and BA/CA (1.09 ± 0.17 vs 1.03 ± 0.16, *P* < 0.05) were significantly higher in Americans than Caucasians. BA–CA and BA/CA were considerably related to lean body mass, BMI, BMI SDS, and X-ray Absorptiometry (DXA) fat mass (all *r* > 0.46, *P* < 0.001) [13].

The previous findings are in accordance with our study showing that there have been accelerated skeletal maturation and significant correlation with lean body mass, BMI, and fat content (*r* = 0.622, 0.435, 0.463; *P* 0.000, 0.001, 0.000, respectively).

The study done by Akridge et al. 2007 showed that the normal, overweight, and obese children had accelerated skeletal age as compared to their chronological age. However, the increase in skeletal age difference was non-significant in all three groups. This distinction is maybe as a result that the population was different in each study. Akridge et al. studied the American population comprising of the whites, whereas this study was done on the Egyptian population. Akridge used Fishman’s method for assessing bone age, whereas in our study Greulich and Pyle method was used (Figs. 1, 2, and 3) [5].

Deb et al. 2017 recruited 150 individuals belonging to age group 10 to 14 years to assess their skeletal maturation using hand-wrist radiographs in late childhood obesity and found that the mean skeletal age difference was more in obese (0.72) than in normal (0.6) and overweight (0.58); however, it had been statistically non-significant *P* = 0.61 and *P* = 0.598, respectively [14]. The main distinction from our study was that the age range was confined to the pubertal period of 10 to 14 years.

Cervical vertebral maturation (CVM) was used widely as another method to hand-wrist radiographs in the assessment of skeletal maturity. Costacurta et al. 2012 conducted a study in 107 individuals belonging to the age group 6 to 12 years; they assessed CVM and dental age, in normal weight, pre-obese and obese patients, using the BMI and the DXA to evaluate their skeletal maturation. They found that no statistically significant differences were determined among the groups regarding their chronological, dental, and skeletal age. Also, they observed no statistically significant differences in the obese subjects as for chronological and skeletal-dental age (*P* = 0.09) [15]. Mack et al. 2013 found that CVM and dental age were more advanced in subjects with increased BMI percentiles [16].

Duplessis et al. 2016 found weak significant correlations between BMI percentile and CVM (*r* = 0.157, *P* < 0.05); higher BMI percentiles correlated with higher CVM stages [17].

In the study by Giuca et al. 2012, 50 white subjects were selected both hand wrist and CVM were accustomed to assess skeletal age. In the normal group, they had a mean delayed skeletal maturation of 2.2 ± 3.1 months. This was similar to this study which showed a mean age difference is − 0.175 ± 0.32 years. Also, 25 obese individuals had a mean accelerated skeletal maturation of 11.8 ± 11.4 months [18]. This was similar to this study which also showed that there was accelerated skeletal maturation.

When skeletal age difference was correlated between the male and female, the females tended to have greater skeletal age differences (0.07) than males (− 0.13); however, it had been non-significant (*P* = 0.138). This is contrary to the study by Sadeghianrizi, 2005 who found that the skeletal age difference was lesser in females than in males which may ensue to a shorter peak of the pubertal spurt for females [19].

Neeley and Gonzales, 2007 expressed that “the orthodontic therapy can be affected by obesity”, given the probability for obese patients to show an irregular pubertal development, due to the hormonal changes associated with obesity, a different bone metabolism; leading to changes in growth and development, and specific craniofacial features; increased mandibular length, shorter upper face height [20]. However, knowing the stage is not sufficient to work out the timing of skeletal maturation accurately, particularly in girls, who show a more precocious maturation and a shorter developmental peak than do boys [19].

Several studies have explored the link between sexual maturity and increased body mass index. When the majority of evidence suggests that there is a significant correlation between the increased BMI and early sexual maturity in females; however, that relationship in boys is still questioned [21]. It has been suggested that those with higher childhood BMI tend to have an earlier onset of puberty [22]. While other investigators have indicated a reverse relationship exists between BMI and pubertal onset in boys [21].

In our study, no significant difference in the Tanner stage in females; this may be due to small sample size; however, in males, there is a significant difference between obese and control groups (*P* value 0.02).

Our results came contradictory to Kaplowitz et al. 2001 who reported that in white girls, BMI is significantly associated with early puberty [23]. Also, a correlation was found between age at adiposity rebound and age at menarche [24].

A study done by Silventoinen et al. 2008 showed that high childhood BMI was associated with an earlier pubertal growth spurt and a strong genetic factor has existed. Growth during puberty was therefore strictly genetically controlled, and these genetic factors also clarified why early maturing children had higher BMI through childhood in this study [25].

In line with our results, the latest research was done by Aksglaede et al. 2009 exploring the connection between pre-pubertal BMI and pubertal onset, evaluated by age at onset of pubertal growth spurt, height velocity peak, growth and puberty in obese children revealed that the heavier children, the earlier they reached puberty [26]. Also, Lee et al. 2016 conducted a study comprised of 3872 subjects to determine whether overweight and obesity were correlated with differences in the timing of puberty in the US boys and found that evidence of earlier puberty for overweight compared with normal boys [27].

Several studies have been done to evaluate the efficacy of using total body fat content as an alternative to BMI as it is the most frequently used index for the classification of overweight-obesity [28]. Our study demonstrates that there was a significant positive correlation between bone age and total body fat content (*r* = 0.463, *P* value = 0.00), a significant correlation between bone age and lean body mass (*r* = 0.622, *P* value 0.00); however, there is a significant negative correlation between bone age and percentage of lean body mass.

A multiple linear regression analysis was performed to detect the influence of clinical and biochemical variables on the degree of advanced skeletal maturation. It was revealed that there was one significant predictor for bone age and directly related to it, which is the lean body mass (with OR = 0.129; 95% CI 0.049–0.210, *P* = 0.002). For our knowledge, we are the first to identify this relation.

Whereas the other confounders (BMI, puberty, total body fat content, and lipid profile) showed non-significant relation to the outcome; this was agreed upon by Kim et al, 2017 who investigated these relations on fifty-three obese children and adolescents (age range, 7–15 years; 32 male and 21 female patients). They revealed that obesity and puberty were not associated with advanced skeletal maturation. BMI SDS was not associated with advanced skeletal maturation in multivariate regression analyses [29].

Although assessment of fat content % by bioelectrical impedance is easy, non-invasive, many studies reported that BIA-derived BF can be biased by multiple variables such as distinctions between study and reference population, skin temperature, skin blood flow, or nutritional status [30]. Limitations of bioelectrical impedance assessment are in morbidly obese patients who have an elevated quantity of extracellular water and total body water, which can overestimate fat-free mass and underestimate fat mass. Central body fat will lead to overestimate the percentage of fat-free mass and underestimate the percentage of fat mass in overweight and obese adults [31].

As the lipid levels were high among obese children; therefore, obesity is considered a risk factor for hypercholesterolemia, and screening obese children for hypercholesterolemia should be considered. Our study showed no statistical difference in the level of cholesterol, LDL or triglycerides between the obese and non-obese group (*P* > 0.05) (cholesterol 170.47 ± 49.22 vs 157.97 ± 16.85 mg/dl; TG 111.77 ± 45.15 vs 103.13 ± 15.11 mg/dl in obese and control children, respectively). However, it was found that mean serum HDL level 39.02 ± 8.69 in the patient group to 49.17 ± 10.68 in the healthy group which was found that those results were statistically significant (*P* value = 0.000).

## Conclusions

Adiposity, as measured by BMI, body fat content %, accelerates growth, puberty, and skeletal maturation. The mean skeletal age difference was greater in the obese group as compared to the control group that suggested accelerated skeletal development in the obese group. It is important to assess skeletal maturity in growing patients to determine the best timing for orthopedic and orthodontic treatment around the growth spurt*.*

## Data Availability

The datasets used and/or analyzed during the current study are available from the corresponding author on reasonable request.

## Abbreviations

BA: Bone age
BF%: Body fat percentage
BMI: Body mass index
CA: Chronological age
CVM: Cervical vertebral maturation
DXA: Dual X-ray absorptiometry
HDL: High-density lipoprotein
LDL: Low-density lipoprotein
*r*: Pearson correlation coefficient
SDS: Standard deviation score
SPSS: S-plus statistics software
